# Pituitary Adenylate Cyclase Activating Polypeptide Has Inhibitory Effects on Melanoma Cell Proliferation and Migration *In Vitro*

**DOI:** 10.3389/fonc.2021.681603

**Published:** 2021-09-20

**Authors:** Tibor Hajdú, Patrik Kovács, Emese Zsigrai, Roland Takács, Judit Vágó, Sinyoung Cho, László Sasi-Szabó, Dániel Becsky, Aniko Keller-Pinter, Gabriella Emri, Kálmán Rácz, Dora Reglodi, Róza Zákány, Tamás Juhász

**Affiliations:** ^1^Department of Anatomy, Histology and Embryology, Faculty of Medicine, University of Debrecen, Debrecen, Hungary; ^2^Department of Family Medicine, Seoul National University Hospital, Seoul, South Korea; ^3^Department of Pediatrics, Faculty of Medicine, University of Debrecen, Debrecen, Hungary; ^4^Department of Biochemistry, Faculty of Medicine, University of Szeged, Szeged, Hungary; ^5^Department of Dermatology, Faculty of Medicine, University of Debrecen, Debrecen, Hungary; ^6^Department of Forensic Medicine, Faculty of Medicine, University of Debrecen, Debrecen, Hungary; ^7^Department of Anatomy, PTE-MTA PACAP Research Team, Szentagothai Research Center, Medical School, University of Pécs, Pécs, Hungary

**Keywords:** PACAP receptors, melanocyte, melanoma, proliferation, migration, invasion

## Abstract

Pituitary adenylate cyclase activating polypeptide (PACAP) is an endogenous neuropeptide which is distributed throughout the body. PACAP influences development of various tissues and exerts protective function during cellular stress and in some tumour formation. No evidence is available on its role in neural crest derived melanocytes and its malignant transformation into melanoma. Expression of PACAP receptors was examined in human skin samples, melanoma lesions and in a primary melanocyte cell culture. A2058 and WM35 melanoma cell lines, representing two different stages of melanoma progression, were used to investigate the effects of PACAP. PAC1 receptor was identified in melanocytes *in vivo* and *in vitro* and in melanoma cell lines as well as in melanoma lesions. PACAP administration did not alter viability but decreased proliferation of melanoma cells. With live imaging random motility, average speed, vectorial distance and maximum distance of migration of cells were reduced upon PACAP treatment. PACAP administration did not alter viability but decreased proliferation capacity of melanoma cells. On the other hand, PACAP administration decreased the migration of melanoma cell lines towards fibronectin chemoattractant in the Boyden chamber. Furthermore, the presence of the neuropeptide inhibited the invasion capability of melanoma cell lines in Matrigel chambers. In summary, we provide evidence that PACAP receptors are expressed in melanocytes and in melanoma cells. Our results also prove that various aspects of the cellular motility were inhibited by this neuropeptide. On the basis of these results, we propose PACAP signalling as a possible target in melanoma progression.

## Introduction

Cutaneous melanocytes are pigment producing dendritic cells that are found in hair follicles and in the stratum basale of the epidermis in human skin. Epidermal melanocytes are in special functional relationship with the surrounding keratinocytes (1). Melanocytes produce melanin pigment granules and transfer them to keratinocytes *via* cytocrine mechanism. Melanin then accumulates around the nuclei of keratinocytes to protect them from DNA-damaging solar ultraviolet radiation. Besides photoprotection, skin coloration and thermoregulation are among the other key melanocyte functions (2). Melanoblasts, early precursors of melanocytes, originate from neural crest and exhibit intense motility and migration (1). After arrival to the epidermis, melanoblasts differentiate to (epidermal or hair follicle) melanocytes or to melanocyte stem cells (1). Differentiated epidermal melanocytes come under the control of keratinocytes. Cell-cell contacts (*via* E-cadherin) between melanocytes and keratinocytes play a key role in regulation of melanocyte proliferation and behaviour with soluble factors being less important, a phenomenon that has been investigated both *in vivo* and *in vitro* (3). Although differentiated melanocytes are settled in a specific milieu and their distribution throughout the body is uniform in every individual (4), varying melanocyte densities in different anatomical parts of the body (5) and wound pigmentation (6) are examples that melanocytes can retrieve at least poor proliferative and/or motile abilities even in an adult.

In consequence of sunburn or local inflammation caused by certain chemicals melanocyte behaviour can change dramatically. Subsequent release of cytokines, such as interleukin-2, can alter the melanocyte-keratinocyte relationship and that induces uncontrolled proliferation eventually (7). Malignant melanoma of the skin is an aggressive neoplastic lesion that originates from epidermal melanocytes (8). The tumour has very poor prognosis once metastases appear, since melanoma cells are highly resistant to any kind of conventional chemotherapy or irradiation (9). Cutaneous melanoma only makes 4% of all skin tumours but is responsible for 80% of skin tumour related death due to metastasis formation (10). High mortality rates and increasing incidence highlight (11) the importance of understanding melanocyte and melanoma biology.

Pituitary adenylate cyclase activating polypeptide (PACAP) was first extracted from ovine hypothalamus as a 38-aminoacid neurohormone (PACAP 1-38) (12) and another biologically active form (PACAP 1-27) was later identified (13). PACAP 1-38 has a short life span (14) due to quick degradation by dipeptidyl peptidase 4 (DPP4). PACAP is an evolutionarily conserved neuropeptide and it is a member of the vasoactive intestinal peptide (VIP) – secretin – growth hormone releasing hormone – glucagon superfamily. Three G-protein coupled receptors have been identified for PACAP binding: VPAC1 and VPAC2 bind VIP and PACAP with equal affinities, while PACAP type I (PAC1) receptor is considered as the receptor with highest affinity to PACAP (15). PACAP has been described in a wide range of regulatory processes of the nervous system and peripheral organs that involve differentiation, cell division, cell cycle and cell death (16–20). It also appears as a neurotransmitter or endocrine regulator in the cardiovascular, respiratory, urogenital, digestive, skeletomuscular and immune systems (15, 21, 22). PAC1 receptor activation can trigger several signalling cascades leading to pleiotropic effects and serving as explanation for how PACAP plays an important role in preventing harmful effects and various kinds of stress (23–27).

Contradictory PACAP effects have been reported in the field of oncology (28). Functions of PACAP are controversial in different tumours as it can inhibit the migration of certain tumour cells such as glioblastoma cells (29) or reduce the tumour growth in cervical cancer (30). On the other hand, it induces tumour growth in neuroendocrine tumours (31) or increases proliferation of osteosarcoma derived UMR-106 (32) and astrocytoma cells (33). Furthermore, dose and time dependent manners of PACAP effects have been described in prostate cancer and neuroblastoma, respectively (34). Low concentrations of PACAP enhanced proliferation of neuroblastoma cells, while cell differentiation was observed at high doses (35). Prostate cancer cells showed cell growth after short-term PACAP treatment, while long-term PACAP administration resulted in differentiation and phenotype switch (34).

Although the effects of PACAP have been identified in sweat glands (36), and in keratinocytes (37, 38), no data can be found on melanocytes. Moreover, the presence of PACAP receptors has been studied in different tumours such as colon, ovarian, breast, lung, prostate, liver or pancreatic cancers (28, 34), but no information can be found related to melanoma. Therefore, the goal of our present study is to examine the presence of PACAP receptors in melanocytes and melanoma cells *in vivo* and *in vitro*. Furthermore, we aimed to investigate the effects of PACAP on human epidermal melanocytes and melanoma cell lines of different stages *in vitro*.

In the present work, we provide evidence that PACAP receptors are expressed by normal and malignant cutaneous pigment cells deriving from two different stages of malignancy and PACAP signalling inhibits proliferation and motility of melanoma cells.

## Material and Methods

### Human Skin and Cutaneous Melanoma Tissue Samples

Normal human skin tissue samples were collected from cadavers (post-mortem 3 days) with the assistance of the Forensic Medicine Department (approved by the Ethics Committee of University of Debrecen, under licence number 3244-7/2011). Tissue samples were washed in phosphate buffered saline (PBS) three times and fixed in 4:1 mixture of absolute ethanol and 40% formaldehyde. Then samples were dehydrated in ascending alcohol row and embedded in paraffin.

Histological blocks of human melanoma tissue samples from both male and female patients with different depths (2.8 mm, 3.1 mm, 8 mm, 8.6 mm depth) were collected from the Department of Dermatology, University of Debrecen (approved by the Ethics Committee of University of Debrecen, under licence number 9555-2/2017/EKU).

### Immunohistochemistry Reactions and HE Stainings of Histological Samples

**Figure 1:**
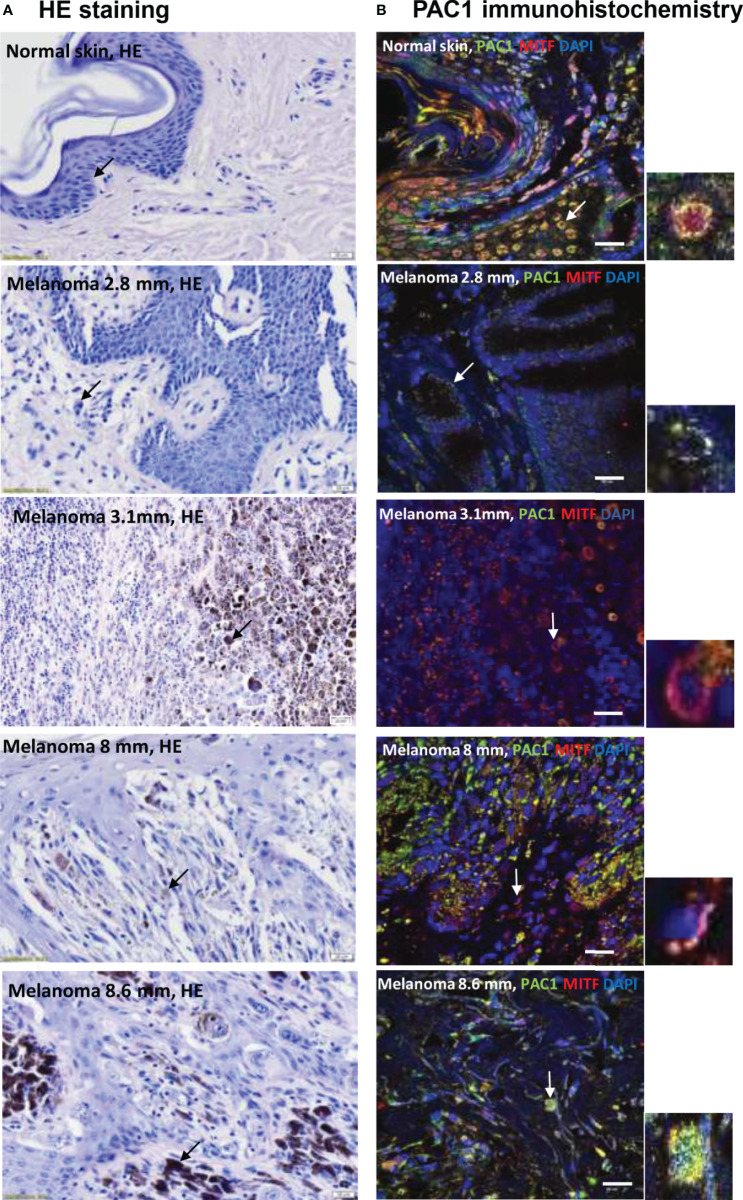
**(A)** HE staining of tissue samples from human skin and melanoma lesions with different depths (2.8 mm, 3.1 mm, 8 mm, 8.6 mm depth). The arrows show pigment containing cells: melanocytes in the basal layer of the epidermis and melanoma cells in the tumorous environment. The original magnification of these images was 40×. **(B)** Immunohistochemical reactions demonstrate the colocalization of anti-PAC1 and the pigment cell specific anti-MiTF antibodies in melanocytes and melanoma cells. The arrows show the PAC1 and MiTF double positive cells magnified in the inserts. The original magnification of these images was 60×. Scale bar 20 µm.

Five-μm thick serial sections were made of normal skin and melanoma tissue samples. After removal of paraffin samples were rehydrated and PBS supplemented with 1% bovine serum albumin (BSA, Amresco LLC, Solon, OH, USA) was applied to block nonspecific antibody binding sites at 37°C for 1 h. Then samples were incubated with polyclonal anti-PAC1 antibody produced in rabbit (Sigma-Aldrich, St. Louise, MO, USA), at a dilution of 1:500 in PBS with 0.1% Tween 20 (PBST, Amresco LLC, Solon, OH, USA), at 4°C overnight. On the following day after washing three times with PBS, biotinylated anti-rabbit antibodies produced in goat were added onto the samples (Vector Laboratories, Burlingame, CA, USA), at a dilution of 1:1000 in PBST, at room temperature for 2 hrs. After washing, tissues were incubated with the second primary antibody, polyclonal anti-microphthalmia-associated transcription factor (MiTF) antibody (Abcam, Cambridge, UK) produced in rabbit, at a dilution of 1:500 in PBST, at 4°C, overnight. On the third day the biotinylated antibody labelling the anti-PAC1 antibody was visualized with Streptavidin Alexa Fluor 488 conjugate, while the MiTF antibody was visualized with anti-rabbit Alexa Fluor 555 secondary antibody (Life Technologies Corporation, Carlsbad, CA, USA) at a dilution of 1:1000 in PBST. Tissue samples were mounted in Vectashield Hard Set mounting medium (Vector Laboratories, Burlingame, CA, USA) containing DAPI (4′,6-Diamidino-2-phenylindole dihydrochloride) for nuclear DNA staining. For testing PAC1 antibody specificity human brain was used (39) (**Supplemetary Figure 1**).

Light microscopical analysis on serial sections stained with haematoxylin-eosin (HE) (Sigma-Aldrich, St. Louise, MO, USA) provided morphological proof to our immunohistochemistry reactions. Following rehydration of the sections, the HE staining protocol was carried out according to the instructions of the manufacturer.

Results of the immunohistochemical reactions were evaluated by an Olympus FV3000 confocal microscope (Olympus Corporation, Tokyo, Japan) using a 60x PlanApo N oil-immersion objective (NA: 1.42) and FV31S-SW software (Olympus Corporation, Tokyo, Japan). Z image series of 1-μm optical thickness were recorded in sequential scan mode. For excitation 488 and 543 nm laser beams were used. The average pixel time was 4 μsec. Images of Streptavidin Alexa Fluor 488, Alexa Fluor 555 and DAPI were overlaid by using Adobe Photoshop version 10.0 software. Photomicrographs of HE stained samples were taken by a DP74 camera (Olympus Corporation, Tokyo, Japan) on an Olympus BX53 microscope (Olympus Corporation, Tokyo, Japan).

### Isolation and Culturing of Primary Human Epidermal Melanocytes

Primary human epidermal melanocytes were isolated and cultured based on methods with modifications described by Godwin et al. (40). Briefly, we used human, juvenile foreskin samples immediately after surgery to isolate melanocytes. The study was approved by the Ethics Committee of University of Debrecen, under licence number: 5011-2018. Following initial sterilization samples were put in 10 mg/mL dispase II (Gibco, Gaithersburg, MD, USA) dissolved in Hank’s balanced salt solution (Gibco, Gaithersburg, MD, USA) overnight to allow gentle separation of the epidermis and dermis. This was followed by trypsinization of the epidermal tissues by 500 µg/mL trypsin/EDTA (Sigma-Aldrich, St. Louise, MO, USA) and centrifugation at 450 × g, for 15 mins, at 4°C. Pellets were resuspended in RPMI-1640 medium (Sigma-Aldrich, St. Louise, MO, USA) and plated in T75 flasks (Eppendorf, Hamburg, Germany). Maintenance of the primary melanocyte culture required RPMI-1640 medium supplemented with melanocyte specific mitogens. Mitogens were applied in the following concentrations: 5 μL/mL 40 μM TPA (12-O-tetradecanoylphorbol 13-acetate; also known as PMA; Sigma-Aldrich, St. Louise, MO, USA), 5 μL/mL 40 nM CT (cholera toxin; Sigma-Aldrich, St. Louise, MO, USA), 2 μL/mL 5 μM ET1 (endothelin-1; Bachem, Bubendorf, Switzerland) and 2 μL/mL 5 μg/mL SCF (stem cell factor; Invitrogen, Carlsbad, CA, USA) per 5 mL RPMI medium. Cells of the culture underwent thorough light microscopical verification. Melanocytes were cultured at 37°C in the presence of 95% air and 5% CO_2_ atmosphere and 80% humidity.

### Human Melanoma Cell Lines

Commercially available stable cell lines were obtained from ATCC (ATCC^®^ CRL-1661™, Manassas, VA, USA). Human cutaneous melanoma cell line A2058 was established from lymph node metastasis of an amelanotic melanoma of a male patient, while WM35 was derived from non-metastasizing superficial spreading melanoma of a female patient. Cells were cultured in RPMI-1640 culture medium (Sigma-Aldrich, St. Louise, MO, USA) supplemented with 10% foetal bovine serum (Gibco, Gaithersburg, MD, USA), 4.1 g/L glucose, 2 mmol/L L-glutamine (Gibco, Gaithersburg, MD, USA), penicillin (100 units/mL) and streptomycin (100 µg/mL). Cells were incubated at 37°C in the presence of 95% air and 5% CO_2_ atmosphere and 80% humidity in 25 cm^2^ flasks until approximately 70% confluence. Both cell lines were routinely screened by polymerase chain reaction (PCR) for possible *Mycoplasma* infection.

### Application of PACAP

PACAP 1-38 at 100 nM (stock solution: 100 μM; dissolved in sterile distilled water) and PACAP 6-38 at 10 µM (stock solution: 10 mM dissolved in distilled water) as a specific antagonist were used in our experimental setup. Untreated (control) and PACAP treated experimental groups were established. PACAP treatment started at 50-60% confluence stage and lasted for 48 hrs with medium changed and application renewed at the 24^th^ hour.

### RT-PCR Analysis

Melanoma and melanocyte cell cultures were washed three times with physiological NaCl dissolved in Trizol (Applied Biosystems, Foster City, CA, USA), and after the addition of 20% RNase free chloroform (Sigma-Aldrich, St. Louise, MO, USA), samples were centrifuged at 10,000×*g* for 15 min at 4°C. Samples were incubated in 500 µL RNase-free isopropanol at –20°C for 1 h then, then total RNA was dissolved in nuclease free water (Promega, Madison, WI, USA) and stored at –70°C. The assay mixture for reverse transcriptase reaction contained 2 µg RNA, 0.112 µM oligo(dT), 0.5 mM dNTP, 200 units of High Capacity RT (Applied Bio-Systems, Foster City, CA, USA) in 1× RT buffer. DNA was transcribed at 37°C for 2 hrs.

Amplifications of specific cDNA sequences were carried out using specific primer pairs that were designed by Primer Premier 5.0 software (Premier Biosoft, Palo Alto, CA, USA) based on human nucleotide sequences published in GenBank and purchased from Integrated DNA Technologies, Inc. (IDT; Coralville, IA, USA). The specificity of custom-designed primer pairs was confirmed *in silico* by using the Primer-BLAST service of NCBI (http://www.ncbi.nlm.nih.gov/tools/primer-blast/). Nucleotide sequences of forward and reverse primers and reaction conditions are shown in **Table 1**. Amplifications were carried out in a programmable thermal cycler (Labnet MultiGene™ 96-well Gradient Thermal Cycler; Labnet International, Edison, NJ, USA) in a final volume of 11 μL (containing 0.5 μL forward and reverse primers [0.4 μM], 0.5 μL dNTP [200 μM], and 5 units of Promega GoTaq^®^ DNA polymerase in 1× reaction buffer) as follows: initial denaturation at 95°C, 2 mins, followed by 35 cycles (denaturation, 94°C, 1 min; annealing at optimised temperatures as given in **Table 1** for 1 min; extension, 72°C, 90 sec) and then further extension at 72°C, 10 mins. PCR products were analysed by horizontal electrophoresis in 1.2% agarose gel containing ethidium bromide (Amresco LLC, Solon, OH, USA) at 120 V constant voltage. Signals were developed with a gel imaging system (Fluorchem E, Protein Simple, San Jose, CA, USA). Results were normalised to the internal control (GAPDH expression).

**Table 1:** Table of primer pairs and PCR reaction conditions.

Gene	Primer	Nucleotide sequence (5’→3’)	GenBank ID	Annealing temperature	Amplimer size (bp)
**prepro** **PACAP**	sense	CCA GAG GAA GAG GCG TAC (231-248)	**NM_001099733.2**	54°C	176
	antisense	AGC ACT TTG CGG TAG GC (406-390)			
**DPP4**	sense	GGA AGT CAT CGG GAT AG (1767-1783)	**NM_001935.4**	50°C	259
	antisense	ATC ATT CAC GCT GCT GT 2025-2009)			
**PAC1**	sense	TCA TCC TTT GTC GCT TCC (807–824)	**NM_001199637.2**	53°C	170
	antisense	GAC GGC CTT ACA TTC CAC (976–959)			
**VPAC1**	sense	CCA TTG CCT GTG GTT TG (857-873)	**NM_004624.4**	54°C	344
	antisense	CAG CCA GAA GAA GTT AGC C (1200-1182)			
**VPAC2**	sense	CGT TCC CAG ATT TCG TCG (488–505)	**NM_003382.5**	53°C	149
	antisense	GAG GCA CAG AAT TAT GCT TCC (636-616)			
**GAPDH**	sense	CCA GAA GAC TGT GGA TGG CC (740–759)	**NM_002046.5**	54°C	411
	antisense	CTG TAG CCA AAT TCG TTG TC (1150–1131)			

### SDS-PAGE and Western Blot Analysis

Cells were washed in physiological NaCl solution and then harvested. After centrifugation, cell pellets were suspended in 100 μL of RIPA (Radio Immuno Precipitation Assay) homogenization buffer (composed of 150 mM NaCl; 1.0% NP40, 0.5% sodium deoxycholate; 50 mM Tris, 0.1% SDS; pH 8.0) supplemented with protein inhibitors as follows: aprotinin (10 ug/mL), 5 mM benzamidine, leupeptin (10 ug/mL), trypsine inhibitor (10 ug/mL), 1 mM PMSF, 5 mM EDTA, 1 mM EGTA, 8 mM Na-fluoride, 1 mM Na-orthovanadate. All components were purchased from Sigma-Aldrich. Samples were stored at −70°C.

Suspensions were sonicated by pulsing burst for 30 sec, at 40 A by 50 cycles using an ultrasonic homogeniser (Cole-Parmer, Vernon Hills, IL, USA). As total cell lysates were used for western blotting, samples for sodium dodecyl sulphate-polyacrylamide gel electrophoresis (SDS-PAGE) were prepared by the addition of Laemmli’s electrophoresis sample buffer (4% SDS, 10% 2-mercaptoethanol, 20% glycerol, 0.004% bromophenol blue, 0.125 M Tris–HCl; pH 6.8) to cell lysates to set equal protein concentration, and boiled for 10 min at 95°C. 20 µg of protein was separated by 7.5% SDS-PAGE for the detection of VPAC1, PAC1, DPP4 and actin. Separated proteins were transferred to nitrocellulose membranes (Bio-Rad Trans Blot Turbo Midi Nitrocellulose Transfer Packs) by using a Bio-Rad Trans-Blot Turbo system (Bio-Rad Laboratories, Hercules, CA, USA). After blocking in 5% non-fat dry milk in PBST for 1 h, membranes were washed and exposed to primary antibodies overnight at 4°C in the dilution as given in **Table 2**. After washing for 30 mins in PBST, membranes were incubated with horseradish peroxidase-conjugated secondary antibodies, anti-rabbit IgG (Bio-Rad Laboratories, Hercules, CA, USA) in 1:1500, or anti mouse IgG (Bio-Rad Laboratories, Hercules, CA, USA) in 1:1500 dilution. Membranes were developed by enhanced chemiluminescence (Advansta Inc., Menlo Park, CA, USA) according to the instructions of the manufacturer. Signals were developed with a gel imaging system (Fluorchem E, Protein Simple, San Jose, CA, USA). Results were normalised to the actin expression.

**Table 2:** Table of antibodies used in the experiments.

Antibody	Host animal	Dilution	Distributor
**Anti-PAC1**	rabbit, polyclonal	1:500	Sigma-Aldrich, St. Louis, MO, USA
**Anti-VPAC1**	rabbit, polyclonal	1:800	Alomone Labs., Jerusalem, Israel
**Anti-VPAC2**	rabbit, polyclonal	1:600	Abcam, Cambridge, UK
**Anti-DPP4**	rabbit, polyclonal	1:200	Abcam, Cambridge, UK
**Anti-Actin**	mouse, monoclonal	1:10000	Sigma-Aldrich, St. Louis, MO, USA

### Immunocytochemistry

Immunocytochemistry was performed on cells cultured on the surface of coverslips to visualize intracellular localizations of PAC1. Cultures were fixed in 4% paraformaldehyde (Sigma-Aldrich, St. Louise, MO, USA) solution for 1 h and washed in distilled water. After rinsing in PBS (pH 7.4), nonspecific binding sites were blocked with PBST supplemented with 1% BSA (Amresco LLC, Solon, OH, USA) for 30 mins, at 37°C. Samples were then washed again three times in PBS, and cultures were incubated with rabbit polyclonal anti-PAC1 antibody (Sigma-Aldrich, St. Louise, MO, USA) at a dilution of 1:500 in PBST, at 4°C overnight. On the following day after washing three times with PBS, the primary antibody was visualized with anti-rabbit Alexa Fluor 555 secondary antibody (Life Technologies Corporation, Carlsbad, CA, USA) at a dilution of 1:1000 in PBST. Cultures were mounted in Vectashield Hard Set mounting medium (Vector Laboratories Burlingame, CA, USA) containing DAPI for nuclear DNA staining. Reactions were repeated three times and recordings of five different visual fields were examined for each sample.

For investigation of PAC1 subcellular localization fluorescent images were taken with an Olympus FV3000 confocal microscope (Olympus Corporation, Tokyo, Japan) using 60× PlanApo N oil immersion objective (NA: 1.42) and FV31S-SW software (Olympus Corporation, Tokyo, Japan). Z image series of 1-μm optical thickness were recorded in sequential scan mode. For excitation 488 and 543 nm laser beams were used. The average pixel time was 4 μsec. Transmission and fluorescents images were merged to visualize membrane localization. Images of Alexa555 and DAPI were overlaid using Adobe Photoshop version 10.0 software.

### Measurement of Cellular Viability

Through examining activities of mitochondrial reductases, we gain information on cellular viability. A2058 and WM35 cells (5000 cells per well) as well as melanocytes (10000 cells per well) were cultured in wells of 24-well plates. Each experimental group consisted of 12 biological samples and the assays were repeated three times (n=36). After plating, cells were cultured for 1 day, and MTT assays were performed after 48 hrs of PACAP treatment. Then, 20 μL MTT reagent [3-(4,5-dimethylthiazolyl-2)-2,5-diphenyltetrazolium bromide; 25 mg MTT/5 mL PBS] (Sigma-Aldrich, St. Louise, MO, USA) was added to each well. Cells were incubated for 2 hrs at 37°C and following the addition of 500 μL MTT solubilizing solution (10% Triton X-100 and 0.1 M HCl dissolved in anhydrous isopropanol) absorbance values were measured at 570 nm (Chameleon Microplate Reader, Hidex Ltd. Turku, Finland).

### Measurement of Cell Proliferation

Cell proliferation of melanocytes and melanoma cells was determined by measuring DNA contents of the cells by using CyQUANT^®^ Cell Proliferation Assay Kit (Invitrogen, Carlsbad, CA, USA) according to the manufacturer’s instructions. A2058 and WM35 cells (2500 cells per well) as well as melanocytes (5000 cells per well) were cultured in 96-well black-well/clear-bottom plates (Greiner Bio-One, Baden-Württemberg, Germany). Each experimental group consisted of 12 biological samples and the assays were repeated three times (n=36). After plating, cells were cultured for 1 day, and CyQUANT assays were performed after 48 hrs of PACAP 1-38 and 6-38 treatment. First, the remaining media were removed from the wells, and then plates were frozen at −70°C. In the next step the plates were thawed at room temperature, and 200 μL of CyQUANT GR dye/cell lysis buffer mixture was pipetted to each well. Incubation in dark for 5 mins was followed by quantification of fluorescent signals by a Fluorescence Imaging Plate Reader FlexStation^III^ (FLIPR, Molecular Devices, CA, USA) at an excitation wavelength of 485 nm and an emission wavelength of 530 nm.

### Migration Assay

PACAP pre-treated primary melanocytes and melanoma cells were washed twice in PBS, harvested with 0.25% trypsin (Sigma-Aldrich, St. Louise, MO, USA) and after centrifugation, cells were resuspended in RPMI medium. Lower wells of Boyden chemotaxis chamber (Neuro Probe Inc., Gaithersburg, MD, USA) were filled with 1 μL/mL human fibronectin (Sigma-Aldrich, St. Louise, MO, USA) dissolved in PBS and covered with a polycarbonate filter (Neuro Probe Inc., Gaithersburg, MD, USA) containing pores with a diameter of 3 µm (Katona et al., 2016). 50 µL of cell suspension in a density of 2×10^5^ cells/mL was inoculated into the wells on the top of the membrane and the chamber was incubated for 3 hrs, at 37°C in a humidified atmosphere (5% CO_2_–95% air) in the presence of PACAP 1-38 and 6-38. Non-migrated cells were removed from the surface of the membrane and after fixation in methanol, migrated cells were stained with 1% toluidine blue (Sigma-Aldrich, St. Louise, MO, USA) dissolved in water. Membranes were air-dried and mounted with Pertex (Sigma-Aldrich, St. Louise, MO, USA). Absolute cell numbers were counted by light microscopical evaluation, by three independent operators.

### Invasion Assay

Melanoma cells were washed twice in PBS, harvested with 0.25% trypsin (Sigma-Aldrich, St. Louise, MO, USA) and after centrifugation, resuspended in RPMI medium in a density of 2.5×10^5^ cells/mL. Corning^®^ BioCoat™ Matrigel^®^ Invasion Chambers (Corning, Glendale, AZ, USA) were filled with control or PACAP treated melanoma cells. Matrigel^®^ contains pores with a diameter of 8 µm. Fibronectin (Sigma-Aldrich, St. Louise, MO, USA) dissolved in RPMI medium at 1 μL/mL concentration was used as chemoattractant in the lower chambers. Incubation time was 22 hrs in a humidified atmosphere as mentioned above. Cells on the target surface of Matrigel^®^ were fixed in 4% paraformaldehyde for 1 h and then washed three times in PBST. Monoclonal anti-actin antibody (Sigma-Aldrich, St. Louise, MO, USA) produced in mouse was used in 1:5000 dilution to highlight invading cells. The primary antibody was visualized with anti-mouse Alexa Fluor 555 secondary antibody (Life Technologies Corporation, Carlsbad, CA, USA) at a dilution of 1:1000. Images of the reactions were taken with an Olympus FV3000 confocal microscope (Olympus Corporation, Tokyo, Japan) using 60× oil immersion objective (NA: 1.42). The parameters of confocal microscopy were the same as mentioned above. Absolute numbers of invaded cells were calculated by three independent operators in 10 different visual fields in each of the three independent repeats of the experiment (n=15).

### Live Imaging Analysis

Phase contrast time-lapse images were taken for 12 hrs (2 frames/min) at 37°C by CytoSMART™ System for cell culture monitoring (Lonza Bioscience, Basel, Switzerland) and analysed using ImageJ (National Institutes of Health, Bethesda, MD, USA, https://imagej.nih.gov/ij/) and CellTracker (http://celltracker.website/) software. During manual tracking of cells, dying or damaged cells were excluded from the analysis. Total path of migration, the average individual cell speed, the maximum distance, and vectorial distance (i.e., real shift of the cell) values were calculated. Migratory tracks (total paths) of individual cells were transposed to a common origin to generate wind rose plots as described earlier (41).

### Statistical Analysis

Data of MTT and CYQUANT assays, as well as migration and invasion assays are representative of at least three independent experiments; data are mean values. Statistical significance between controls (non-treated cells) and PACAP-treated pigment cells was determined by one-way analysis of variance (ANOVA), followed by Tukey’s HSD *post hoc* test. Threshold for statistically significant differences as compared to control samples was set at *p < 0.05.

## Results

### Presence of PACAP Receptor PAC1 in Melanocytes and Melanoma Cells Detected in Skin Histological Slides

Five-µm-thick serial sections were selected for HE staining (**Figure 1A**) and PAC1 receptor immunohistochemistry reactions on tissue samples of normal skin and melanoma with various depths (2.8, 3.1, 8 and 8.6 mm). HE staining was used to show the position of pigment producing cells in normal skin and in malignant lesions (**Figure 1A**). Melanocytes and melanoma cells were labelled with pigment cell specific anti-MiTF antibodies. In the epidermis of normal skin signals of anti-PAC1 antibodies were detected in keratinocytes and in MiTF-positive melanocytes (**Figure 1B**). As MiTF is a transcription factor its nuclear immunoreactivity was predictable. In several, but not all melanocytes the nuclear MiTF pattern showed colocalization with PAC1 signals. PAC1 immunoreactivity also appeared in the cytoplasm and in the cell membrane, although the latter was often indistinct as clear patterns in the plasma membrane were often covered by intracellular signals. Additionally, definition of membrane signals was less improved due to tight contacts between cells in the tissue. Melanoma cells showed PAC1 immunopositivity independently from the depth and histological type of the tumour (**Figure 1B**). PAC1 signals were detected in most of the nuclei. Similarly to melanocytes, PAC1 was demonstrated in the cytoplasm (**Figure 1B**), but cell membrane expression could not be clearly determined. Expression of VPAC1 and VPAC2 receptors were followed but positive signals were not identified either in tumour-free normal skin or in melanoma-containing samples.

### PACAP Receptor Expression in Human Epidermal Melanocyte and Melanoma Cell Cultures

**Figure 2:**
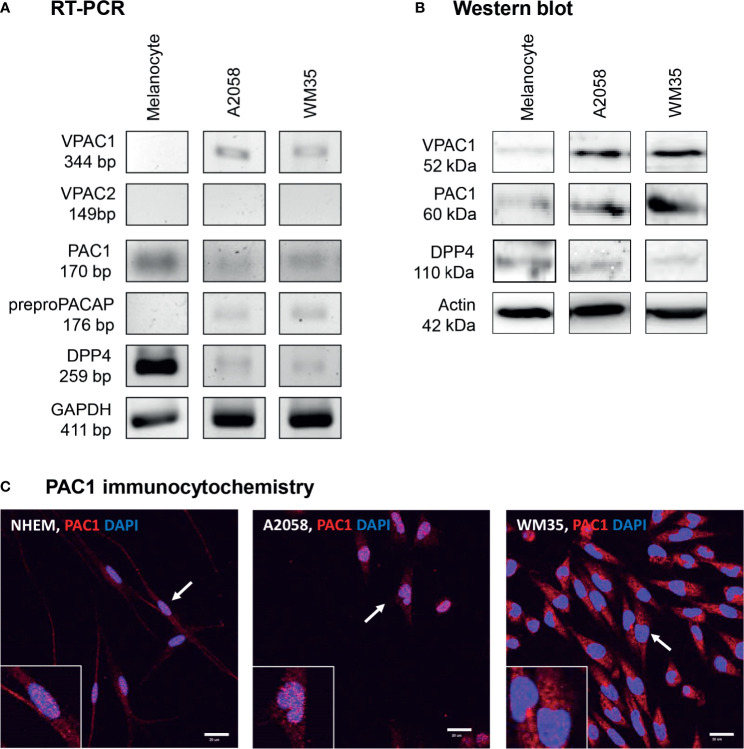
mRNA **(A)** and protein **(B)** expression of PACAP, its receptors and DPP4 in human pigment cells. For RT-PCRs and for western blots GAPDH and actin were used as internal controls, respectively. Images are representative data of three independent experiments. **(C)** Immunocytochemical reactions of PAC1 in pigment cells. The arrows show the positive cells magnified in the inserts. Magnification of the images is 60×. Scale bar: 20 µm.

To investigate the possible effects of PACAP first we examined the presence of PACAP receptors in human epidermal melanocytes isolated and cultured in our laboratory. WM35 cell line was used to represent the radial growth phase of *in situ* melanoma while A2058 cell line isolated from metastatic melanoma was used as an *in vitro* model of aggressive melanoma. mRNA expression of specific PACAP receptor, PAC1 was detected in melanocytes and melanoma cells (**Figure 2A** and **Supplementary Figure 5**). From the non-specific PACAP receptors only VPAC1 mRNA expression was shown and only in melanoma cells (**Figure 2A** and **Supplementary Figure 5**). VPAC2 was not present at mRNA level in the examined pigment cells (**Figure 2A** and **Supplementary Figure 5**). PACAP producing capability was also examined by PCRs through the mRNA expression of preproPACAP. The reactions proved preproPACAP production in WM35 and A2058 cells but not in melanocytes (**Figure 2A** and **Supplementary Figure 5**). DPP4, an enzyme that plays a role in PACAP catabolism, showed strong signals in melanocytes, but weak expression was detected in melanoma samples (**Figure 2A** and **Supplementary Figure 5**). Western blot analysis on PACAP receptors revealed the presence of PAC1 receptor in each pigment cell culture, with no differences in expression (**Figure 2B** and **Supplementary Figure 6**). We found signals of VPAC1 protein expression on the threshold of detection in melanocytes, but strong protein bands were shown in the melanoma cell lines without expression differences (**Figure 2B** and **Supplementary Figure 6**). DPP4 chemiluminescent signals appeared in all cell cultures (**Figure 2B** and **Supplementary Figure 6**). As PAC1 mRNA and protein expression analysis proved results worthy for further investigation, we performed PAC1 immunocytochemistry on our pigment cell cultures. PAC1, the most potent PACAP binding receptor was dominantly present in the cell membrane of *in vitro* melanocytes while in melanoma cells the possible membrane positivity was hidden by diffuse cytoplasmic signals (**Figure 2C**). Membrane signals were revealed by transmission mode in confocal microscopy (**Supplementary Figure 2A**) without positive signals in negative controls (**Supplementary Figure 2B**). Similarly to our immunohistochemistry observations PAC1 was present in the nuclei of melanocytes and melanoma cells (**Figure 2C**).

### PACAP Alters Proliferation but Not the Viability of Melanoma Cells

**Figure 3:**
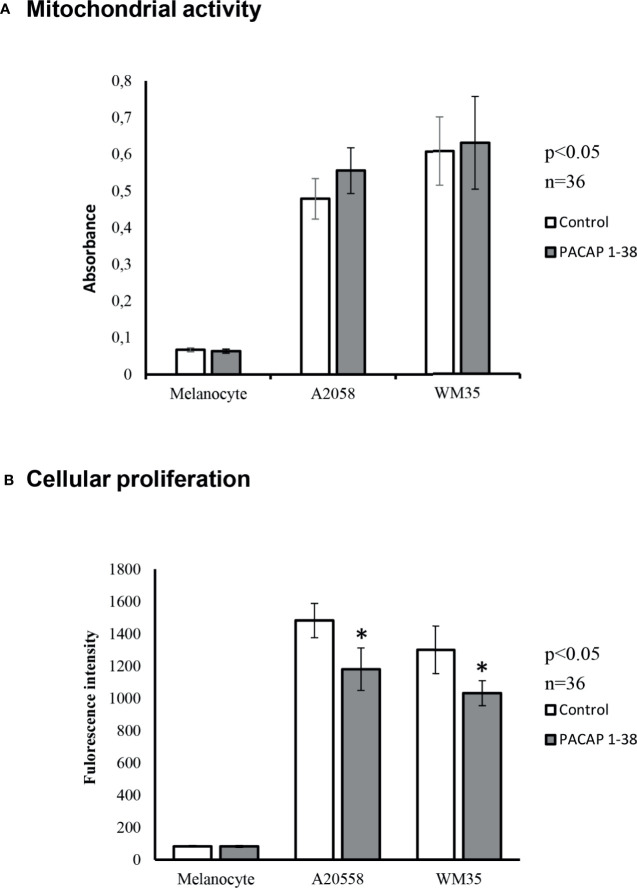
Effects of PACAP administration on mitochondrial metabolic activity (MTT) **(A)** and cellular proliferation (CyQUANT) **(B)** in A2058 and WM35 cell lines and primary melanocytes. Asterisks indicate significant (*p < 0.05) alterations in viability and cell proliferation as compared to the respective untreated control. Statistical significance was determined by one-way analysis of variance (ANOVA), followed by Tukey’s HSD *post hoc* test.

The identification of the PACAP receptors in melanocytes and melanoma cells was followed by the investigation of PACAP’s effects on human pigment cells. MTT assay was performed to check viability changes due to PACAP administration. Cells were plated with similar densities in 24-well plates and the assay started at 90% confluence state. This allowed us to compare results obtained from the different cultures. Mitochondrial activities of melanocytes were lower than in the metabolically active melanoma cells. However, after PACAP treatment significant differences could not be observed in any of the cultures compared to the controls (**Figure 3A**).

Proliferation of pigment cells was examined by CyQUANT assay. With this method, the intensity of fluorescent signals is proportional with the DNA content. Cultured melanocytes, similarly to *in vivo* circumstances, had low proliferative potentials and PACAP addition did not result in significant changes (**Figure 3B**). On the contrary, melanoma cells can be characterized by accelerated proliferation that showed a significant decrease after PACAP 1-38 administration in both melanoma cell lines (**Figure 3B**). However, the PAC1 specific inhibitor, PACAP 6-38 slightly increased the proliferation of melanoma cells (**Supplementary Figure 3**).

### Migratory and Invasive Capabilities of Melanoma Cells Decreased After PACAP Treatment

**Figure 4:**
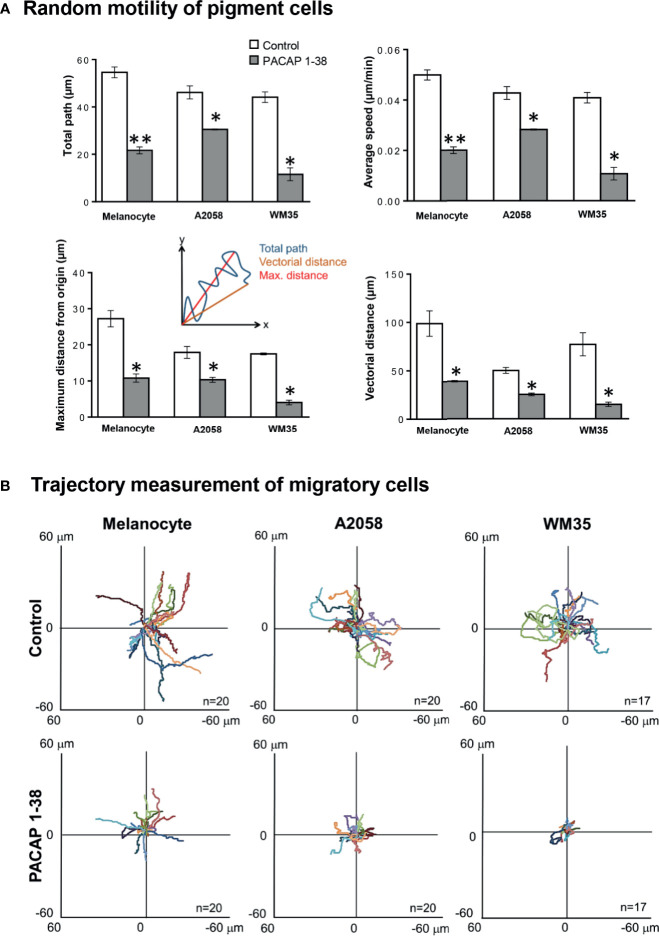
Effect of PACAP treatment on 2-dimensional, random migration of pigment cells. Melanocytes, WM35 and A2058 cells were plated and random migration was imaged for 12 h using time-lapse microscopy following PACAP treatment **(A)**. The migration of the individual cells was tracked, and the total path of migration, average speed of the cells, the maximum distance, and the vectorial distance (real displacement of the cells) were quantified. Quantification of the results is shown, data are reported as means of the independent experiments, n = 3 independent experiments, 82-207 cells/cell line/independent experiment were analysed, error bars represent SEM (*p < 0.05, **p < 0.01). Statistical significance was determined by one-way analysis of variance (ANOVA). Representative wind-rose plots depicting the migration tracks of the individual cells are shown **(B)**. Each coloured line represents the total migration path of a single pigment cell either without or with PACAP 1-38 treatment.

Motility of healthy and pathological pigment cells was monitored by the CytoSMART system. The total way of migration was approximately 50 μm both by melanocytes and melanoma cells (**Figure 4A**). PACAP treatment reduced motility and the total way of migration significantly decreased in every cell culture (**Figure 4A**). Total path of movement, maximal distance of migration from a given origin, vectorial distance value and average speed of cellular movements were higher in primary melanocytes compared to malignant pigment cell lines. Furthermore, vectorial distance proved to be the shortest in A2058 cells, indicating the decreased directionality of these cells. Total path, average speed and maximum distance values were comparable in A2058 and WM35 cell lines; however, WM35 cells were more sensitive to PACAP treatment. PACAP treatment resulted in significant decrease of all parameters in every cell culture (**Figure 4A**), and particularly major alterations were detected in melanoma cells derived from *in situ* melanoma (**Figure 4A**). Taken together, random migration decreased in all cell cultures following PACAP treatments. Next, we shifted the migratory trajectories of the individual cells to a common origin and generated wind-rose plots (**Figure 4B**). Smaller diameter of these wind-rose plots indicate the decreased migration of PACAP treated cells (**Figure 4B**).

**Figure 5:**
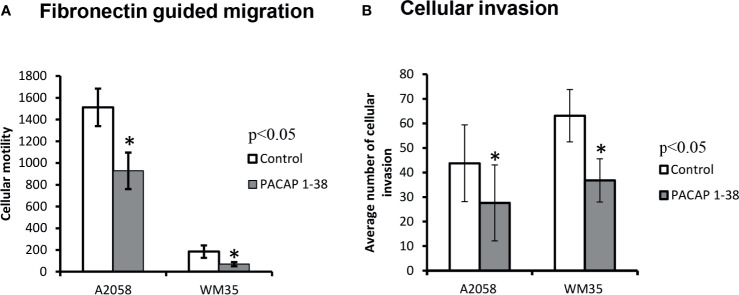
Effects of PACAP on migration **(A)** and invasion **(B)** of melanoma cells. Fibronectin was used as a chemoattractant in both assays. Data represent mean ± SEM of 6 independent wells (migration assay) or 4 independent wells (invasion assay). Results are representative data of 3 independent experiments. Asterisks indicate significant (*p < 0.05) decrease in the number of migrated or invading cells as compared to the respective control. Statistical significance was determined by one-way analysis of variance (ANOVA), followed by Tukey’s HSD *post hoc* test.

Besides the examination of random migration, we also observed in malignant pigment cells the possible changes in fibronectin guided motility as a response to PACAP. We were not able to detect migration in primary melanocytes. Similarly to our previous findings migratory ratios were higher in A2058 cells than in WM35 cells. In the Boyden-chamber, PACAP 1-38 pre-treated melanoma cells showed reduced motility through 3 μm membrane pores, but the rate of decrease was similar in both cell lines (**Figure 5A**). Inhibition of PAC1 receptor increased the migration in WM35 cell lines but it did not significantly alter the migration capability of A2058 cells (**Supplementary Figure 4**). We also observed that melanoma cells can migrate through the pores of the extracellular matrix imitating Matrigel^®^ in a fibronectin chemoattractant driven manner that demonstrates invasiveness. Melanoma cells pre-incubated with PACAP for 48 hrs showed significant decrease in the number of invading cells compared to non-treated, control circumstances (**Figure 5B**).

## Discussion

PACAP, a neuropeptide with a wide functional repertoire, PACAP has been recognised as a tool to fine-tune balances in cellular processes. PACAP has been proven to have important functions throughout the body including the musculoskeletal (20, 39, 42), and central nervous system (43, 44). However, there are no published data available on PACAP related functions in human cutaneous melanocytes and melanoma cells.

Various types of cells in the human skin have been demonstrated to express components of PACAP signalling, such as keratinocytes (37), dermal dendritic cells (45) and Langerhans cells (46). However, cutaneous melanocytes in this context remained unmapped. In our study we demonstrated PACAP production, elimination and PACAP receptor expression in human cutaneous melanocytes. Melanocytes *in vitro* did not show signs of PACAP production as the mRNA of the PACAP precursor was not detected. Perhaps the lack of PACAP precursor transcripts may be attributable to the way the cells are exposed to this neuropeptide. It is plausible to hypothesize that sympathetic nerves are the source of the neuropeptide for melanocytes and potentially for every other PACAP receptor-expressing cell in the skin, rather than an autocrine PACAP signalling loop (45). On the other hand, the expression of DPP4, the enzyme that is responsible for the degradation of PACAP was identified in cultured melanocytes. This suggests that melanocytes possess the necessary tool to regulate PACAP related stimuli, although PACAP is not the only substrate of DPP4.

The analysis on PACAP receptor expression revealed that *in vitro* melanocytes dominantly harbour PAC1 receptors. This finding is especially interesting regarding the fact that PAC1 has the highest affinity to PACAP among its receptors. In histological samples PAC1 immunoreactivity was also observed in MiTF positive melanocytes. In addition to PAC1, VPAC1 and VPAC2 receptors were also monitored. While VPAC1 was weakly expressed as detected by PCRs and western blots, VPAC2 expression did not reach the threshold of detection, suggesting the pivotal role of PAC1 receptors in melanocytes.

Confocal microscopic evaluations provided interesting additional data on subcellular localization of PAC1. The expression of the receptor was observed in the cytoplasm and the cell membrane but surprisingly also in the nuclei of melanocytes. The nuclear presence of classic plasma membrane proteins is not a novel phenomenon. Our laboratory described earlier that NMDA receptor subunits are present in the nuclei of melanoma cells and implied the possession of nuclear localization signals (NLS) by the examined subunits (47). Furthermore, VPAC1 has been reported to be present in the nuclei of *in vitro* breast cancer cells (48). Nuclear PAC1 expression has already been demonstrated in spermatids (49), suggesting the possibility of an intracrine regulation. Moreover, nuclear dimerization and localization of PAC1 receptor has also been confirmed affecting cellular proliferation in CHO cells (50). The mechanism which enables PAC1 to enter the nucleus is not yet clear. It can happen either by lateral diffusion or direct membrane trafficking in the lipid bilayers of the endoplasmic/nucleoplasmic reticulum by means of a NLS that provides importin binding (51). There has been no information published whether any splice variants of PAC1 possess NLS. However, any putative NLS-suspect area of PAC1 needs thorough examination and functional confirmation (52). To explain the nuclear presence of PAC1, we analysed whether RNA, DNA or histone binding sequences were present in the receptor by corresponding protein sequence databases (www.uniprot.org). As this screening carried out negative results, we excluded the possibility that PAC1 influences transcriptional events or chromatin structure *via* direct molecular interactions with the chromatin. Therefore, we presume that PAC1 may act as a nuclear G-protein coupled receptor and intervenes nuclear signalling *via* cyclic adenosine monophosphate (51).

Malignant transformation of melanocytes results in the formation of melanoma (53). To compare our findings on healthy pigment cells with a putative PACAP-signalling of melanoma cells, we also examined PACAP production, elimination and PACAP receptor expression in A2058 and WM35 melanoma cell lines. To confirm the relevance of our findings in normal and pathologic skin we investigated PAC1 receptor expression in melanoma containing skin tissue samples as well. mRNA expression of the PACAP precursor showed strong bands in samples of cultured melanoma cells suggesting that malignant pigment cells, unlike melanocytes can produce PACAP. The need and importance for PACAP in neoplastic pigment cells is further strengthened by the fact that both melanoma cell lines showed weaker expression patterns at mRNA and protein levels for DPP4 in contrast to melanocytes. Interestingly, PAC1 expression in malignant cells was similar to that in melanocytes. Immunopositive signals were visualized in the plasma membrane of A2058 and WM35 cells, but cell contours were not as clearly identified by the expression pattern as in melanocytes. PAC1 also appeared in the nuclei of melanoma cells. Nuclear PAC1 receptor signals were at similar intensities and showed similar distribution in the nucleus in each cell culture, suggesting that nuclear presence of PAC1 does not correlate with malignancy. As we discussed, melanocytes are unable to produce PACAP, but melanoma cells are. Therefore, not only paracrine and autocrine mechanisms, but intracrine signalling is also feasible in malignant pigment cells, given that the receptors are present in the nuclei. Concept and evidence on intracrine stimulation in relation to PACAP has been described in spermatids (49) in support of our findings. Furthermore, PAC1 receptor localization has been discussed in several tissues and it has been proven that the receptor internalization is rapidly induced by PACAP addition in cardiac muscle cells and endosomal localization has also been shown which increased the cardiac neuron excitability (54, 55). PAC1 receptor immunopositivity has been shown on intracellular organelles inducing the activation of RTK signalling (56). VPAC1 expression was also detected in melanoma cells but showed stronger bands in western blots than in melanocytes. PAC1 expression in normal melanocytes and melanoma cells was demonstrated also in skin tissue by immunocytochemistry.

To understand the roles of PACAP in healthy and pathological pigment cells we added the full biological form of the neuropeptide (PACAP 1-38) and PACAP 6-38 as a PAC1 receptor antagonist to our cell cultures and examined potential changes in viability and proliferation. No alterations were found in metabolic activity/viability by MTT assays in any of the cell cultures. PACAP has been published to have ambivalent effects on proliferation. In certain neural cells PACAP is able to enhance, while in other cells it can decrease the rate of cell divisions (57). Specifically in neoplastic cells, extrinsic PACAP has been shown to have opposing effects on proliferation of breast cancer cells (28, 58). In our experiments, we observed reduced proliferation of melanoma cells upon PACAP 1-38 treatments but slight elevation was shown in the presence of the antagonist.

Cellular motility and migration are essential properties for both melanocyte precursors and melanoma cells. Therefore, we examined the effects of PACAP on two-dimensional (2D) migration of cultured melanoma cells and primary melanocytes (59, 60). Random movements of cells in Petri dishes represent migratory capacities; however, this is not in correlation with invasiveness. Influence of PACAP on total distance, average speed, maximum distance from origin and vectorial distance of 2D migration were examined and analysed. The involvement of PACAP in migration has been demonstrated in cerebellar granule cells (61) and the neuropeptide has been reported to have repressive effects on motility in the cerebellar cortex (62). Normally, differentiated cutaneous melanocytes are not expected to migrate at large scale, but melanocyte precursors require intensive mobility to get from the neural crest to the developing skin (1). Interestingly, cultured juvenile melanocytes showed 2D migration parameters similar to those in melanoma cells, suggesting that melanocytes may have or may regain such migratory capabilities *in vitro*. Although we did not examine the roles of PACAP in migratory processes of melanoblast, significant reduction was observed in migratory parameters after PACAP treatments in cultured melanocytes. Administration of PACAP resulted in reduction of migratory parameters in both A2058 and WM35 melanoma cell lines. However, the rate of the reduction in melanocytes and melanoma cells was similar, indicating that the degree of PACAP’s effects on migratory parameters is independent from malignancy. Little is known about the effect of PACAP on motility of other malignant cells, but a similar phenomenon has been described in glioblastoma where PACAP decreased cell migration (63). It is worth to mention that PAC1 receptor has the highest affinity to bind PACAP 1-38, moreover, it has been detected that PACAP 1-38 had PAC1 receptor selectivity over VPAC1 receptor (64). To strengthen our results cells were treated with PACAP 6-38 the specific antagonist of PAC1 receptor and migration assays were also performed. Migration of WM35 cells showed a significant increase in the presence of the antagonist, while the migration capability of A2058 cells did not alter significantly. These results also suggest that the alterations dominantly activate PAC1 receptor which has a direct effect on melanoma motility and proliferation.

Invasiveness is a key neoplastic feature, offering the ability of malignant cells to penetrate healthy tissues and to establish new local tumour cell clusters. Several mutations have been identified in melanoma that affect signalling pathways involved in cellular motility leading eventually to invasion and metastasis formation (65). Therefore, the next step was to examine the effects of extrinsic PACAP on fibronectin-guided migration and invasion of melanoma cells. Melanocytes did not show 3D migration capability. PACAP 1-38 treatments significantly decreased the number of migrating cells in the Boyden chamber and the number of invading cells through the basement membrane imitating Matrigel. There was no difference in the rate of reduction between WM35 and A2058 melanoma cells, suggesting that PACAP-related migration/invasion reduction may be independent from tumour stage. As the inhibition of PAC1 receptor with PACAP 6-38 increased the migration it also suggests a PAC1 receptor mediated motility. The involvement of VIP/PACAP-signalling has been recognised to have anti-tumour effects in glioblastoma cells. Stimulation of VIP/PACAP receptors triggers the cAMP/PKA axis, that reduces migration and invasion of glioblastoma cells *via* the inhibition of the PI3K/Akt and the Shh/GLI1 signalling routes (63). Furthermore, inhibition of the Shh/GLI1 pathway directly, or in combination with the Ras/Akt pathway, decreases melanoma growth (66). This also reduces the chance of recurrence through the repression of growth of the putative melanoma cancer stem cells (66). PACAP has also been reported to have an effect on the MAPK pathway (67, 68), a signalling route that notoriously gains mutations during melanoma progression (69). Taken together, it is likely that extrinsic PACAP can influence three-dimensional (3D) migration of melanoma cells *via* different signalling routes.

Our study revealed that human cutaneous melanocytes and melanoma cells possess PAC1 receptor which enables the cells to evoke intracellular responses upon PACAP stimulation. The unconventional, partly intranuclear localization of PAC1 receptor was a surprising finding but this phenomenon seemed less likely to depend on malignancy. In addition, as melanoma cells can produce PACAP, paracrine, autocrine and intracrine regulatory mechanisms are all possible. Exogenous PACAP reduced proliferation and migration/invasion of melanoma cells. Questions can be raised why neoplastic cells produce a neuropeptide and express its receptors if this neuropeptide has negative effects on the tumour cells. The ambivalence can be resolved with an often-used concept about PACAP, namely that the neuropeptide acts as a fine-tuning regulator of homeostasis during cellular stress situations. From the perspective of neoplastic cells PACAP may provide a sensitive tool to adjust the cellular behaviour towards the adaptation of cells to the stressful challenges during metastasis formation and further progression. As exogenous PACAP decreased proliferation and migration/invasion *in vitro*, it suggests that this neuropeptide could be further investigated to prove its possible function in the inhibition of melanoma progression.

## Data Availability Statement

The original contributions presented in the study are included in the article/**Supplementary Material**, further inquiries can be directed to the corresponding author/s.

## Ethics Statement

The studies involving human participants were reviewed and approved by Ethics Committee of University of Debrecen. The patients/participants provided their written informed consent to participate in this study.

## Author Contributions

Study conception and design: TJ, DR. Foreskin operation and sample collection were established by LS-S. Melanoma and skin sample collection was done by GE and KR. Melanocyte primary cultures were isolated and established by TH, EZ, PK and SC. TH, EZ, PK and SC performed the proliferation, viability and motility assays. Molecular biological reactions were done by JV, RT and TH. Immunohistochemistry and histology were performed by TH, EZ and TJ. Invasion and migration assays were performed by TH and TJ. Cell motility analysis was established by DB and AK-P. Statistical analysis is made by TH. Acquisition of data: TH and JT. Analysis and interpretation of data: TH, TJ, RZ and DR. Participated in drafting the manuscript: TH, RZ, TJ and DR.

## Funding

NKFIK115874, PD109644, K119759, NKFIHFK129190, 135457, PTE AOK Research Grant, MTA-TKI 14016, GINOP-2.3.2-15-2016-00050 “PEPSYS”, New National Excellence Program of the Ministry of Human Capacities (UNKP-16-4-IV.), EFOP-3.6.1.-16-2016-00004 Comprehensive Development for Implementing Smart Specialization Strategies at the University of Pécs, EFOP-3.6.2-16-2017-00008, University of Debrecen (OTKA Bridging Fund) NKFIH K 139396, EFOP-3.6.1.-16-2016-00004 Comprehensive Development for Implementing Smart Specialization Strategies at the University of Pécs (Budapest, Hungary) NAP B KTIA NAP 13-2014-0022 (MTA-PTE NAP B Pain Research Group, identification number: 888819), NAP 2017-1.2.1-NKP-2017-00002 and OTKA-NN 114458. The project is co-financed by the European Union and the European Social Fund. The published work was supported by EFOP-3.6.3-VEKOP-16-2017-00009 co-financed by EU and the European Social Found (HT). This work was supported by the ÚNKP-18-2, the ÚNKP-19-2 and the ÚNKP-20-2 New National Excellence Program of the Ministry of Human Capacities (PK, EZ). Szodoray Lajos and Magyary Zoltán Funds by Hungarian Academy of Science and the European Union and the State of Hungary, co-financed by the European Social Funding. Fund in the framework of TÁMOP 4.2.4 (TJ). The work was supported by the National Research, Development and Innovation Office of Hungary (grant nos: GINOP-2.3.2-15-2016-00040 and NKFI FK 134684) and by the János Bolyai Research Scholarship of the Hungarian Academy of Sciences (AK-P).

## Conflict of Interest

The authors declare that the research was conducted in the absence of any commercial or financial relationships that could be construed as a potential conflict of interest. All authors contributed to the article and approved the submitted version.

## Publisher’s Note

All claims expressed in this article are solely those of the authors and do not necessarily represent those of their affiliated organizations, or those of the publisher, the editors and the reviewers. Any product that may be evaluated in this article, or claim that may be made by its manufacturer, is not guaranteed or endorsed by the publisher.

## Abbreviations

PACAP, pituitary adenylate cyclase activating polypeptide; VIP, vasoactive intestinal polypeptide; VPAC, vasoactive intestinal peptide receptor; PAC1, pituitary adenylate cyclase-activating polypeptide type I receptor; DPP4, dipeptidyl peptidase 4; PBS, phosphate buffered saline; PBST, PBS with 0.1% Tween 20; MiTF, microphthalmia-associated transcription factor; DAPI, 4′,6-diamidino-2-phenylindole dihydrochloride; HE, haematoxylin and eosin; PCR, polymerase chain reaction; RT-PCR, reverse transcription followed by PCR; mRNA, messenger ribonucleic acid; SDS-PAGE, sodium dodecyl sulphate-polyacrylamide gel electrophoresis; MTT, 3-(4,5-dimethylthiazolyl-2)-2,5-diphenyltetrazolium bromide; NLS, nuclear localization signal; ECM, extracellular matrix; MMP, matrix metalloproteinase; 2D, two-dimensional, 3D, three-dimensional.
