# Supplementary material for: Pituitary Adenylate Cyclase Activating Polypeptide Has Inhibitory Effects on Melanoma Cell Proliferation and Migration In Vitro
Source: Front Oncol. 2021 Sep 20;11:681603. doi: 10.3389/fonc.2021.681603 (PMC8488289; doi:10.3389/fonc.2021.681603)
Supplement: Supplementary file 1 [file DataSheet_1.pdf]

PAC1 western blot positive control

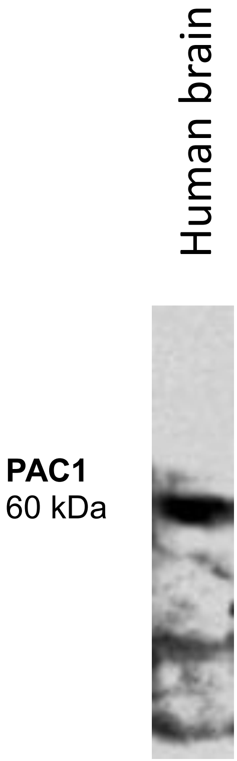

Supplementary Figure 1.
Protein expression of PAC1 receptor in human brain.

A. PAC1 immunocytochemistry in TM images

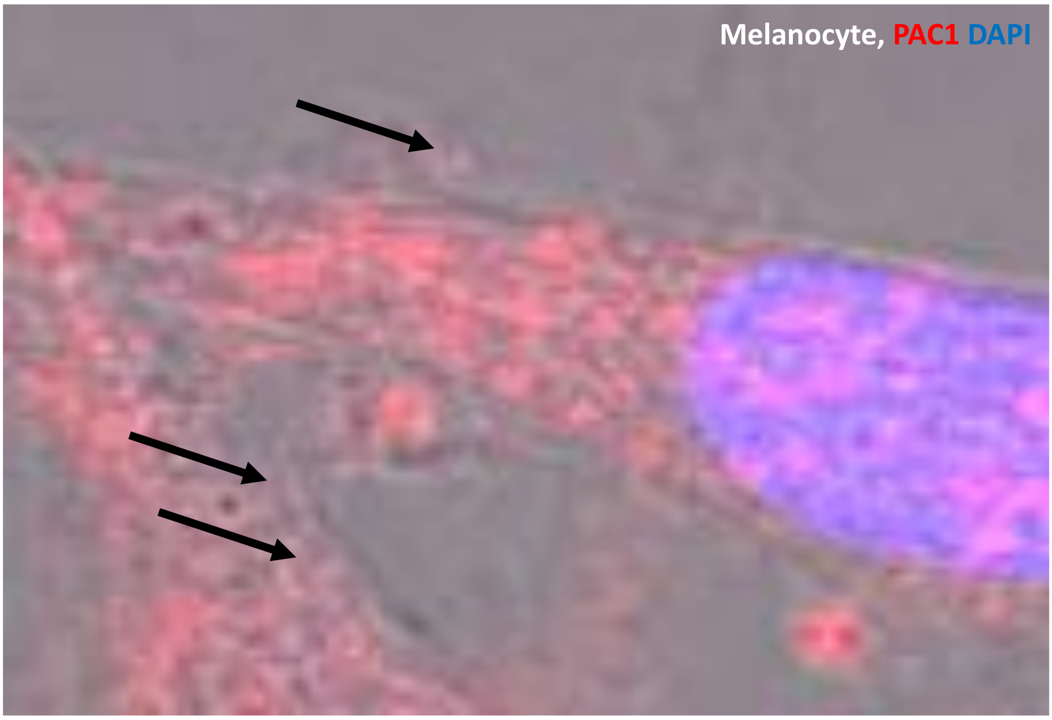

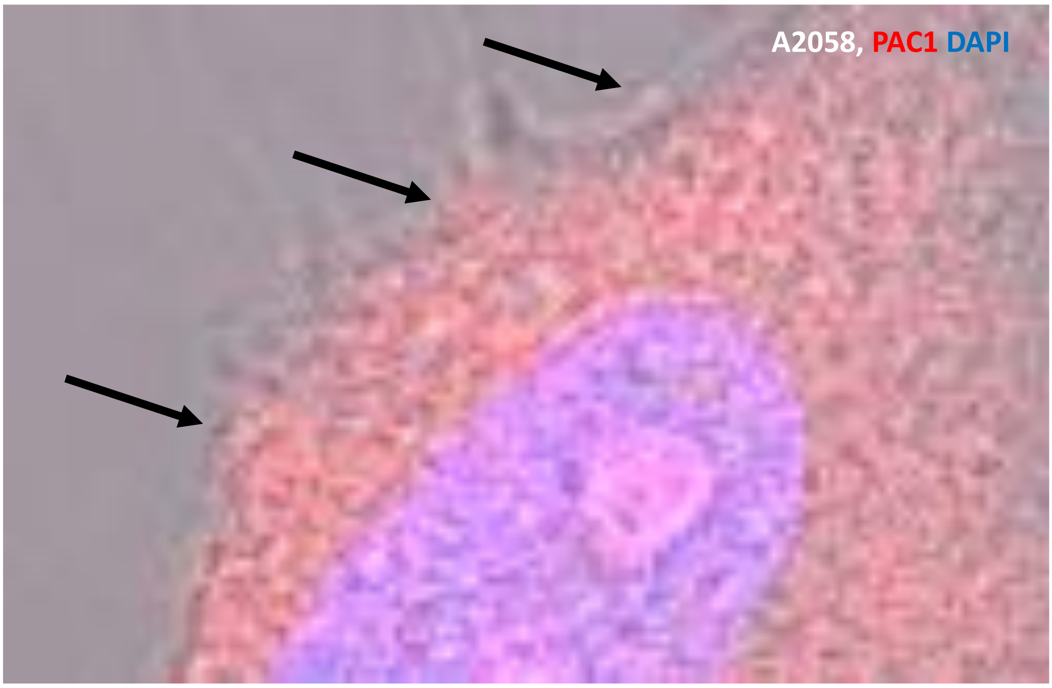
A2058, PAC1 DAPI

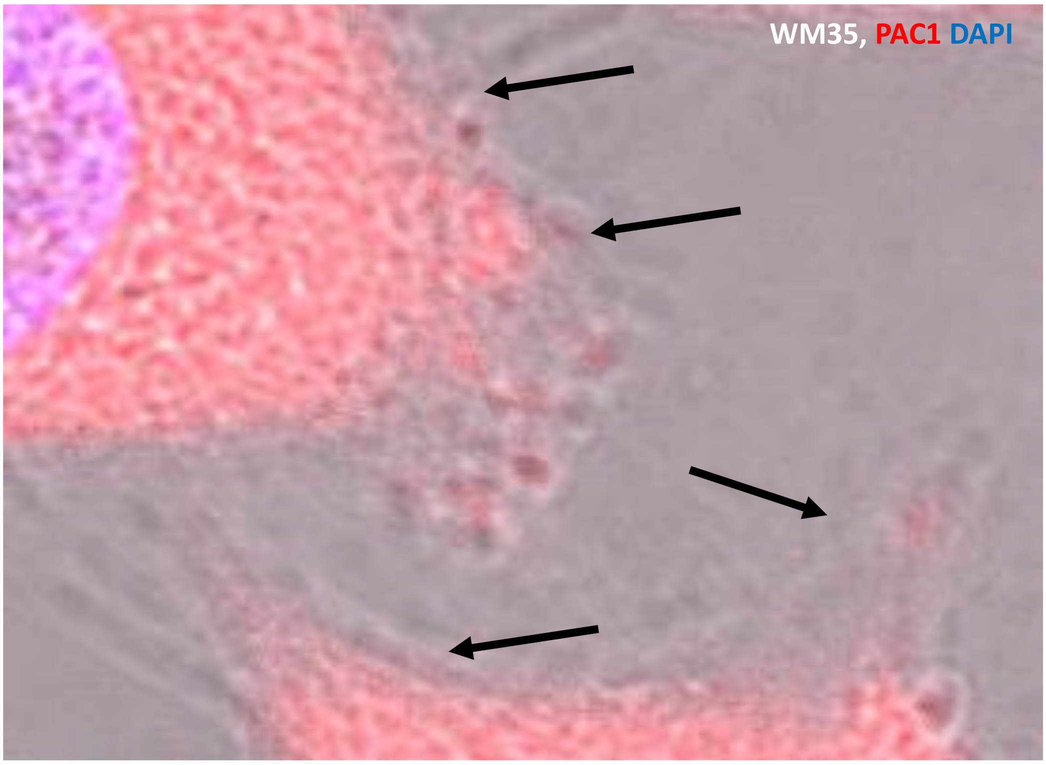
WM35, PAC1 DAPI

B. PAC1 immunocytochemistry negative control

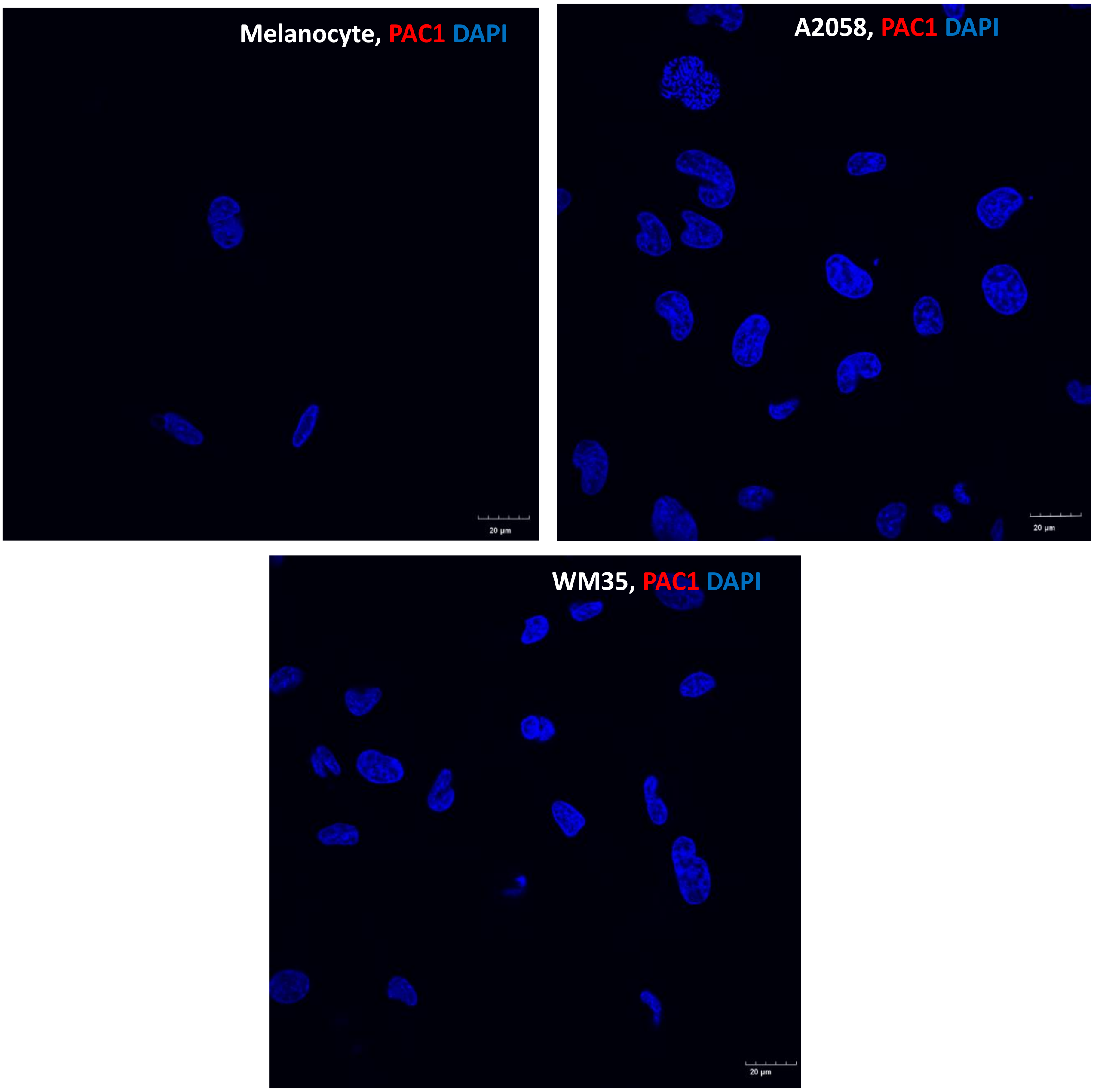

Supplementary Figure 2. A. Immunocytochemical reactions of PAC1 in pigment cells. The arrows show the positive cells magnified in the membrane in transmission mode (TM). B. No-primary antibody control. Original magnification of the images is 60×.

Cellular proliferation during PACAP 6-38 administration

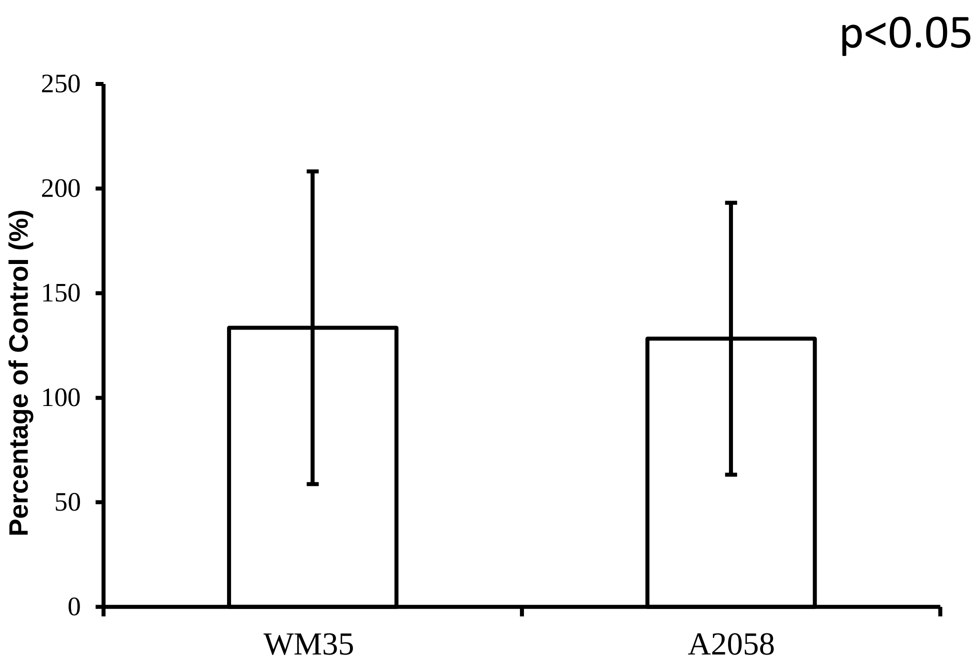

Supplementary Figure 3. Effects of PACAP 6-38 administration on cellular proliferation (CyQUANT) in A2058 and WM35 cell lines. Asterisks indicate significant (*$p<0.05$) alterations in cell proliferation as compared to the respective untreated control. Statistical significance was determined by one-way analysis of variance (ANOVA), followed by Tukey's HSD post hoc test.

Fibronectin guided migration

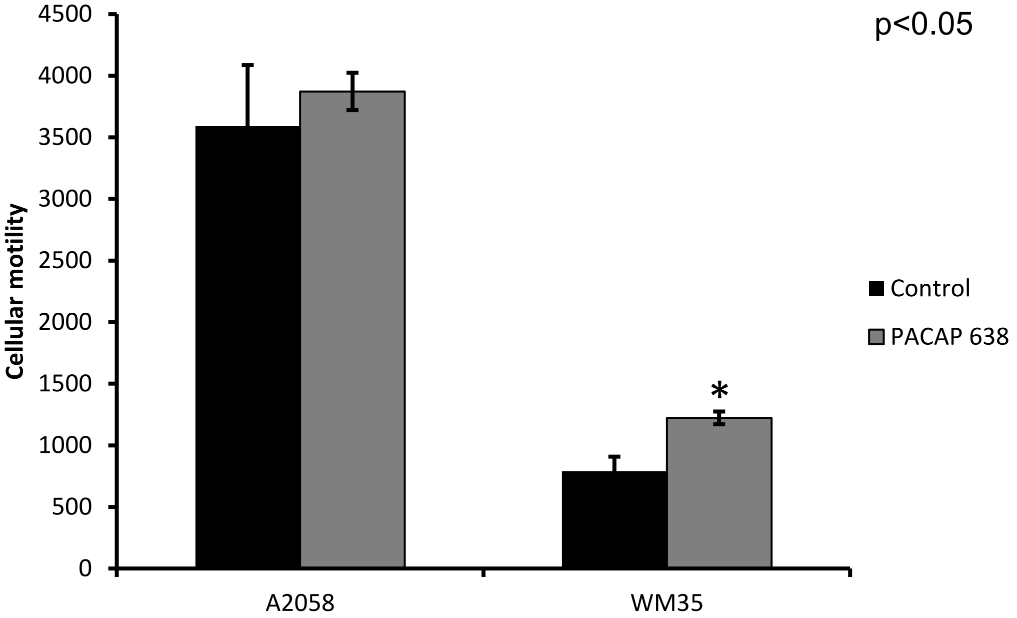

Supplementary Figure 4. Effects of PACAP 6-38 on migration of melanoma cells. Fibronectin was used as a chemoattractant. Data represent mean ± standard error of the mean of 6 independent wells. Results are representative data of 3 independent experiments. Asterisks indicate significant (*p<0.05) decrease in the number of migrated cells as compared to the respective control. Statistical significance was determined by one-way analysis of variance (ANOVA), followed by Tukey's HSD post hoc test.

Gel photos of RT-PCR reactions

RT-PCR

PAC1

170 bp

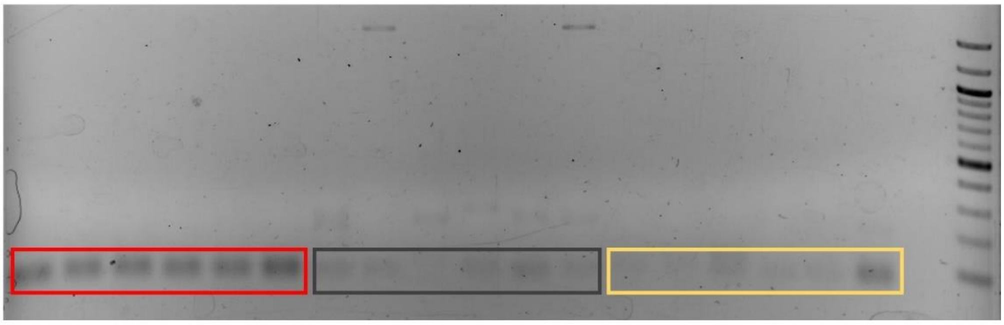

Melanocyte, + PACAP 3 WM35, +PACAP 3, A2058, +PACAP 3

RT-PCR

VPAC1

344 bp

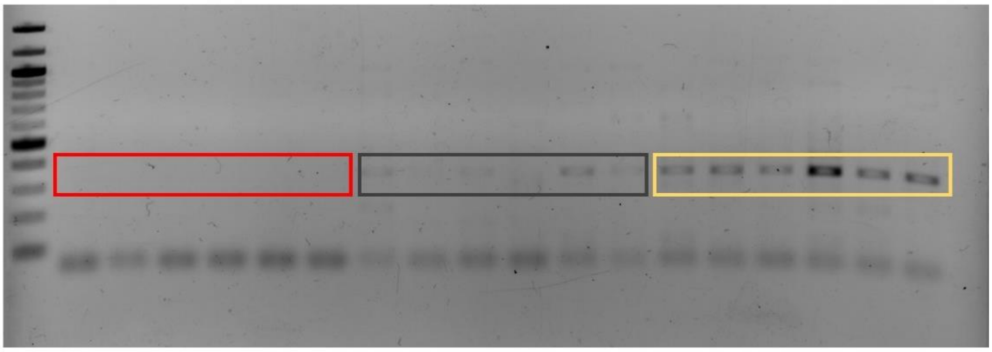

Melanocyte, + PACAP 3 WM35, +PACAP 3, A2058, +PACAP 3

RT-PCR

VPAC2

149bp

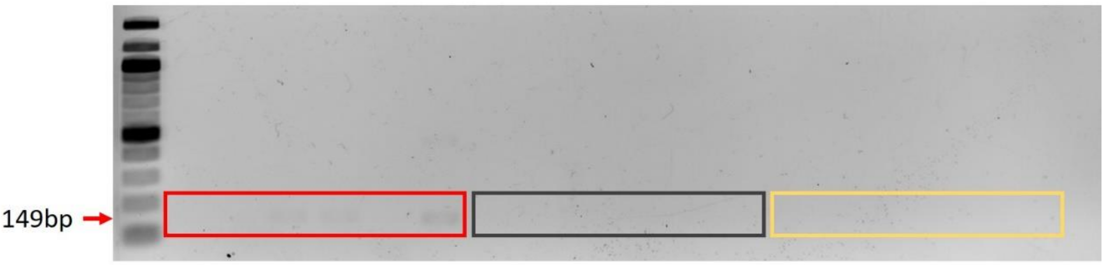

Melanocyte, + PACAP 3 WM35, +PACAP 3, A2058, +PACAP 3

RT-PCR

DPP4

259bp

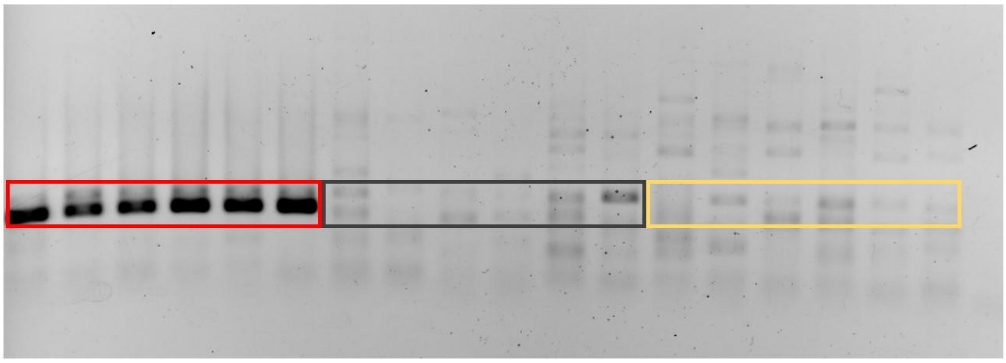

Melanocyte, + PACAP 3 WM35, +PACAP 3, A2058, +PACAP 3

RT-PCR

GAPDH

411 bp

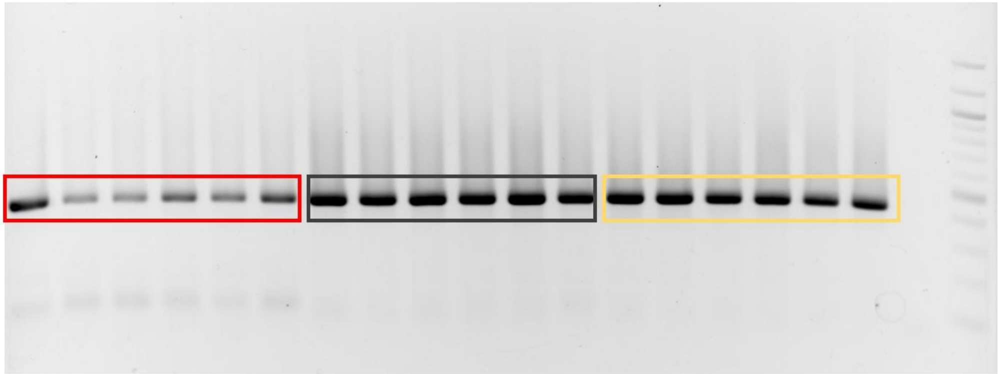

Melanocyte, + PACAP 3 WM35, +PACAP 3, A2058, +PACAP 3

Supplementary Figure 5. Complete gel photos of RT-PCR reactions of PAC1, VPAC1, VPAC2, DPP4 and GAPDH.

Membranes of Western blot reactions

Western blot

PAC1 (56-58 kDa)

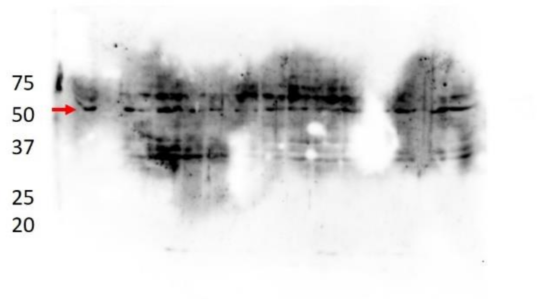

A2058 3 control, WM35 4 control, A2058 3 +PACAP, WM35 4 +PACAP

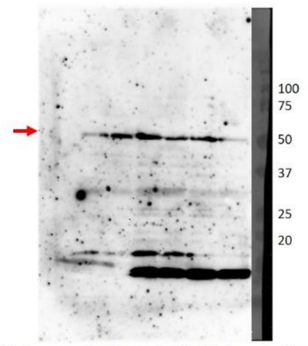

Melanocyte 3 control, Melanocyte +PACAP 3

Western blot

VPAC1 (61 kDa)

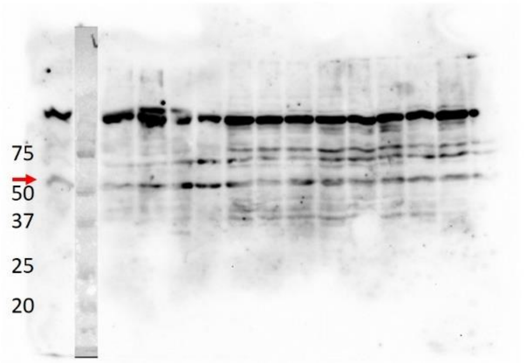

A2058 3 control, WM35 4 control, A2058 3 +PACAP, WM35 4 +PACAP

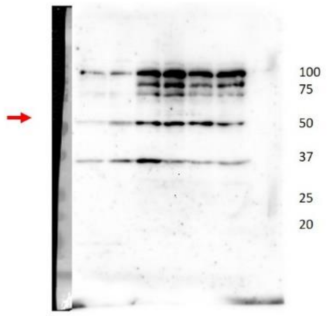

Melanocyte 3 control, Melanocyte +PACAP 3

Western blot

VPAC2 (50-60 kDa)

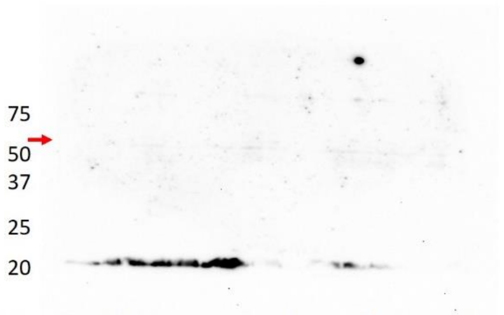

A2058 3 control, WM35 4 control, A2058 3 +PACAP, WM35 4 +PACAP

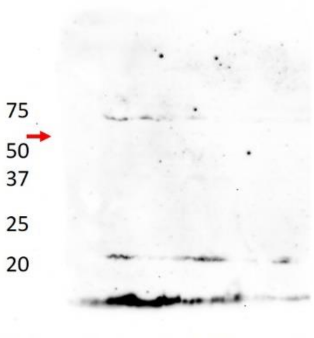

Melanocyte 3 control, Melanocyte +PACAP 3

Western blot

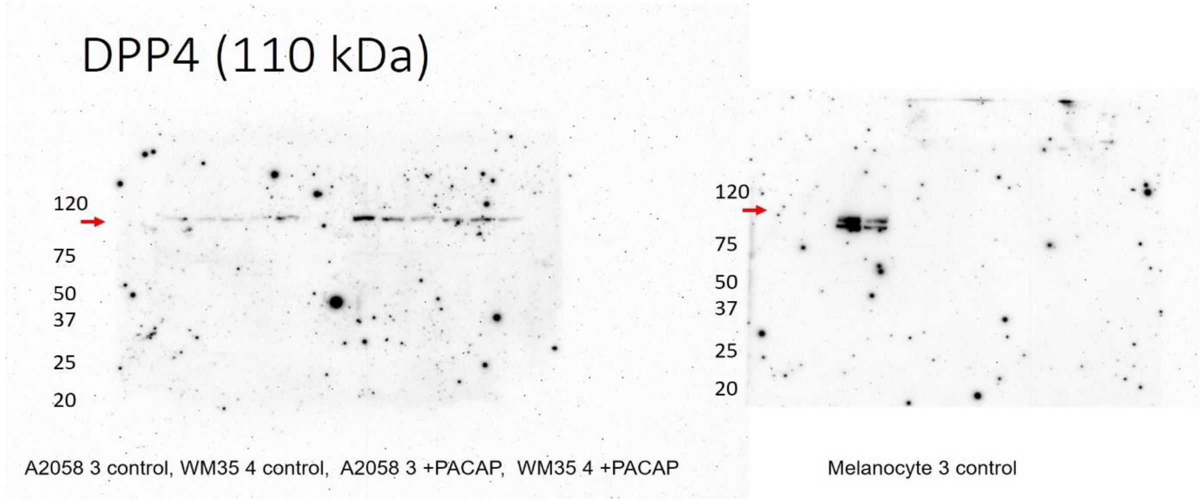

Western blot

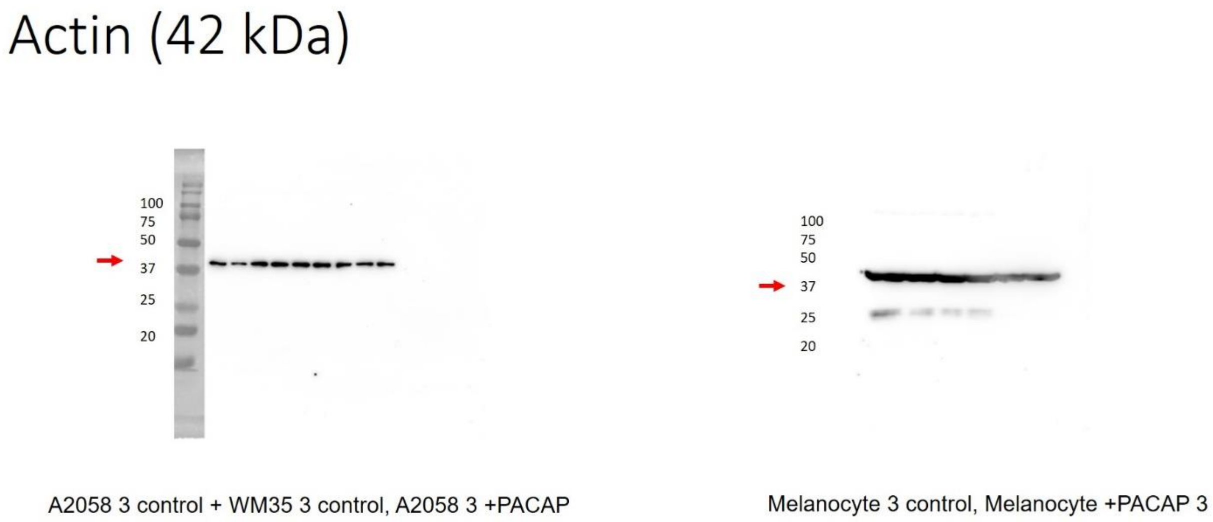

Supplementary Figure 6. Complete membrane photos of Western blot reactions of PAC1, VPAC1, VPAC2, DPP4 and Actin.
